# Efficacy of Commercially Available Nutritional Supplements: Analysis of Serum Uptake, Macular Pigment Optical Density and Visual Functional Response

**DOI:** 10.3390/nu12051321

**Published:** 2020-05-06

**Authors:** Richard A. Bone, Pinakin Gunvant Davey, Betzabe O. Roman, David W. Evans

**Affiliations:** 1 College of Arts, Science and Education, Florida International University, Miami, FL 33199, USA; bone@fiu.edu (R.A.B.); broma009@fiu.edu (B.O.R.); 2 College of Optometry, Western University of Health Sciences, Pomona, CA 91766, USA; 3 Guardion Health Sciences Inc, San Diego, CA 92128, USA; devans@vectorvision.com

**Keywords:** lutein, zeaxanthin, *meso*-zeaxanthin, macular pigment optical density, PreserVision, Lumega-Z, contrast sensitivity

## Abstract

**Purpose:** To compare the change in serum carotenoids, macular pigment optical density (MPOD) and visual function with the intake of two commercially available nutritional supplements. **Methods:** Participants were given a 24-week supply of a lipid-based micronized liquid medical food, Lumega-Z™ (LM), containing 28 mg of the macular carotenoids lutein (L), zeaxanthin (Z) and *meso*-zeaxanthin (MZ), or given PreserVision™ AREDS 2 Formula (gel-caps; PV) containing 12 mg of the macular carotenoids L and Z, but no reported MZ. Serum levels of L, Z and MZ were obtained at baseline and after 12 weeks. Macular pigment optical densities (MPOD) and visual function were assessed at baseline and after 24 weeks. **Results:** Average blood serum concentrations of L, Z and MZ in the two groups at baseline were similar. The increases in L, Z and MZ were 0.434, 0.063 and 0.086 µmol/L vs. 0.100, 0.043 and 0.001 µmol/L, respectively, in the LM vs. PV group. From baseline to week 24, average MPOD in the LM-group increased by 0.064 from 0.418 to 0.482, whereas in the PV-group, it was essentially unchanged (0.461 to 0.459;). Although log-contrast sensitivity was improved in all groups under three conditions (photopic, mesopic and mesopic with glare), the change in log-contrast sensitivity was not statistically significant. Conclusion: Despite only a 2.3-fold higher carotenoid concentration than PV, LM supplementation provides approximately 3–4-fold higher absorption, which leads to a significant elevation of MPOD levels.

## 1. Introduction

The macular pigment is composed of three lipid-soluble carotenoids: lutein, zeaxanthin, and *meso*-zeaxanthin [1,2]. These are responsible for the fovea’s yellow pigmentation and are densely concentrated within the axons of photoreceptors and inner plexiform layers at the center of the macula [2,3,4]. With the exception of *meso*-zeaxanthin, the two carotenoids lutein and zeaxanthin can only be acquired through dietary intake and cannot be synthesized de novo within the eye [2,5,6]; sources include many vegetables, particularly spinach and corn, and also egg yolks. [2,7]. *Meso*-zeaxanthin is a biochemical isomer of lutein and zeaxanthin, is present across the macula, and is synthesized from lutein in the eye or acquired through supplementation [2,5,8]. Numerous studies have shown that oral supplementation of these carotenoids can quickly improve their levels in the serum. However, there is a significant lag in increase in retinal carotenoids once the serum carotenoids have plateaued [9]. 

Carotenoid concentrations and levels in the body have been associated with numerous physiological functions, and their levels correlated with various disease states. Macular carotenoids are quantified by the macular pigment optical density (MPOD) and are associated with maintaining retinal health and optimal visual performance [2,3,7,10], suggesting the level of MPOD is important. Research suggests the carotenoids protect the retina, specifically the macula, via two methods: 1) they act as a filter against blue light and 2) they reduce oxidative stress in retinal tissue [2,7,11]. Due to the MPOD absorption spectrum in the blue part of the visible spectrum, 400–500 nm (peak ~ 460 nm), macular pigment reduces the amount of blue light that reaches the photoreceptor cells, which may indeed provide some improvement in visual function. 

Age-related macular degeneration (AMD) is a leading cause of vision loss, with 196 million individuals suffering with AMD worldwide, which was predicted to increase to 288 million in by the year 2020 [2,3,12]. Retinal levels of carotenoids, particularly MPOD levels, having protective capabilities have led researchers to investigate the role of MPOD levels in the development of eye diseases, such as AMD [11,13,14]. A correlation has been demonstrated between low dietary carotenoid intake and increased risk of developing AMD within Western populations [2,11,13,14]; thus, low levels of MPOD may be related to the progression of AMD [2,15]. Prior reports have shown that oral supplementation of carotenoids can increase MPOD levels [9,16,17,18,19,20,21]; however, the degree to which the increase in MPOD changes visual performance has varied between studies and requires further investigation to establish an optimal carotenoid supplement and delivery methods for individuals with AMD. A recent study, the Age-Related Eye Disease Study 2 (AREDS-2), evaluated the effects of a carotenoid multivitamin supplement containing lutein, zeaxanthin, omega-3 fatty acids, and other antioxidants, in subjects with AMD [22]. The AREDS-2 formulation used in the study, and that is currently commercially available, is PreserVision™, a soft-gel capsule (Bausch and Lomb inc, Bridgewater, NJ, USA). The study concluded that the AREDS-2 oral supplement with carotenoids proved to be efficacious in attenuating the progression of macular degeneration in some patients to advanced AMD [22]. 

One school of thought is that carotenoid vitamin therapies typically administered by conventional soft-gel formulas may be limited due to their efficacy of absorption (EOA) rates [23,24,25,26], prompting researchers to investigate more efficacious delivery systems with improved absorption. Recently, nanoemulsion carotenoid therapies have been investigated in studies with similar micronized delivery agents. Lumega-Z™ (Guardion Health Sciences Inc, San Diego, CA, USA) is a micronized-lipid-based, liquid supplement and a medical food that is designed to restore and maintain the macular pigment in individuals who are at risk of AMD. There are no head-to-head comparisons of the two commercially available products PreserVision™ (PV) and Lumega-Z™ (LM). As a first step, the aims of this study were to evaluate the benefits of PV and LM in an “optimal” environment in ocular-healthy individuals with no known systemic health issues of absorption. This was done by assessing: 1) serum uptake of the carotenoids, 2) changes in MPOD, 3) changes in contrast sensitivity function (CSF), and 4) changes in visual acuity, with intake of LM and PV supplements. Care must be taken in interpreting the results of such a study because, as noted below, PV and LM provide different amounts of the retinal carotenoids in the recommended daily doses.

## 2. Materials and Methods

### 2.1. Subject Demographics

A total of 30 subjects were planned to be recruited in the study, making a cohort of 15 in the LM group and 15 in the PV group. However, one subject from the LM group was disqualified by the investigator early in the study due to non-compliance with taking the supplement. Thus, one additional subject was recruited for the LM group, making the total participants examined for the study to 31. These were recruited from the students, faculty and staff population at Florida International University. Subjects were required to be in good health and able to follow instructions. Efforts were made to include members of minority populations. The study excluded individuals who were smokers since smoking could interfere with the absorption of carotenoids; women who were pregnant (or planning pregnancy) because the absorbed carotenoids could be passed on to the fetus; diabetics because diabetic retinopathy would be an issue; those with hypertension because we do not know how the products might interfere with medication; those with gastrointestinal disorders (not including gastric reflux) because our study requires normal absorption of the products in the gastrointestinal tract. The study was approved by the Institutional Review Board (IRB) of Florida International University. Subjects signed an IRB-approved informed consent form and the study complied with IRB regulations as well as the Declaration of Helsinki (IRB-17-0322-CR01). 

There were 15 participants in each group. The mean age and standard deviation (SD) was 24.6 (9.5) and 29.8 (19.4) in the LM group and PV group respectively, and these were not significantly different (independent samples t-test *p* = 0.36). The male-to-female ratio was not significantly different between the groups (f-test *p* = 0.98). The self-reported ethnicity for the majority of the participants was Hispanic (75%); two participants in the LM group were Caucasian, whereas four participants reported Caucasian and two participants reported Asian ethnicity in the PV group.

The subjects were split into two supplement groups: one group received LM (LM-group) and another received PV (PV-group). Subjects were asked not to modify their diets, and an assessment of their normal dietary intake of carotenoids was not included in this study. Two subjects, one the in LM group and one in the PV group, withdrew from the study after the 12-week follow-up visit. Four additional subjects, two in the LM-group and two in the PV-group, did not complete the study at the 24-week period. 

### 2.2. Supplements

The LM supplement is a multivitamin liquid formulation that provides a daily dose of 15 mg of lutein, 10 mg of *meso*-zeaxanthin, and 3 mg of zeaxanthin along with vitamins and minerals. Subjects were instructed to take the recommended dose (0.75 FL OZ) with a meal once per day throughout the supplementation period. The PV supplement is in the form of gel caps and provides a daily dose of 10 mg of lutein and 2 mg of zeaxanthin together with a few vitamins and minerals. Thus, the LM group received ~ 2.3 × the amount of total carotenoid compared with the PV group. For complete lists of ingredients in LM and PV see Appendix A). Subjects in the PV group were given a seven-day pill organizer to aid compliance, and both groups were given a schedule for future visits including dates for receiving refills. 

### 2.3. Study Duration

Blood serum levels of carotenoids generally respond rapidly to carotenoid supplementation, reaching a plateau after about 8 weeks [9], so a period of 12 weeks was allotted to assess the serum uptake of carotenoids. However, because the macular pigment responds more slowly, we adopted a 24-week supplementation period. Past studies have shown that, during such a time period, significant changes in MPOD can generally be anticipated [9].

### 2.4. Study Related Measurements

The following measurements were performed on all participants: 1) serum levels of carotenoids, lutein, zeaxanthin and *meso*-zeaxanthin were measured at baseline and week 12 post intake of the supplements; 2) visual acuity was measured using an ETDRS chart at baseline and at week 24 (final visit); 3) contrast sensitivity under various conditions—a) photopic, b) mesopic, and c) mesopic with induced glare—was also measured at baseline and at week 24; 4) MPOD measurements were performed at baseline and at week 24. 

#### 2.4.1. Visual Acuity Measurements

Visual Acuity was measured using the ETDRS chart-based acuity test (CSV-1000E, VectorVision, Greenville, OH, USA). Participants were tested at 8 feet and instructed to read sequences of letters within a specified row, using the study eye (usually right) only. The ETDRS test contained rows of five (5) letters, positioned accurately according to established log of Minimum Angle of Resolution (logMAR) score values. The chart luminance was calibrated to 85 cd/m^2^, as recommended for photopic conditions. The logMAR measurements for each subject were obtained in accordance with ETDRS chart scoring protocols.

#### 2.4.2. MPOD Measurements

MPOD was determined in the study eye of each subject using the mapcat SF™ [27], a heterochromatic flicker photometer used in a customized mode (cHFP). (“Customized” refers to a procedure for optimizing the flicker frequencies for each subject.). The subject’s field of view was a circular stimulus subtending a visual angle of 1.5° and provided with crosshairs for central fixation. The stimulus alternated between blue and green wavelengths provided by LEDs. While blue light is strongly absorbed by the macular pigment, green light is only weakly absorbed. Thus, the stimulus generally appears as a flickering turquoise color due to mismatched luminances. For the customizing procedure, the subject viewed the stimulus with the green light switched off. Starting with a high flicker frequency (~ 45 Hz), the subject was asked to reduce the frequency until flicker was just perceived. This is the critical fusion frequency, CFF. Next, the frequency was set to 2/3 of the CFF, and, with the blue and green lights on, the subject adjusted the intensity of the blue light until flicker stopped or was minimized. This occurs at equiluminance of the blue and green lights. Frequency adjustments were made only if the subject reported either a range of no flicker (frequency too high), or failure to eliminate flicker (frequency to low). The blue light intensity setting made by the subject indicated the degree of attenuation of blue light, principally by the macular pigment but also by the lens. The latter contribution increases considerably with age.

The test was repeated using a 15° stimulus, again provided with crosshairs for central fixation, and a default frequency set at 5/6 of the CFF. Subjects were now asked to adjust the blue intensity to eliminate flicker in the peripheral part of the stimulus while ignoring any residual flicker at the center. Five repeat measurements were made for each part of the test, and the test was considered acceptable if the standard error in the mean MPOD was less than 0.015. Automatic calculations of MPOD and the standard error, as described in Bone and Mukherjee [27], were performed by the instrument’s microprocessor. The entire test was repeated a second time, and a weighted mean of the MPOD was calculated, together with a standard error using an algorithm published by Olive et al. [28]. 

#### 2.4.3. Contrast Sensitivity Measurements

The VectorVision CSV-1000E was used to measure the contrast sensitivity function (CSF). Measurements were obtained via standardized patient procedures described in prior publications [29,30,31]. In brief, the chart-based test presented a series of sine-wave targets of varying spatial frequency (3, 6, 12, and 18 CPD). Four rows of achromatic sine-wave targets were rear-illuminated (mean luminance 85 cd/m^2^) and self-calibrated for photopic conditions, each corresponding to one spatial frequency [29,30]. Across each row, vertical target pairs were illuminated with eight different contrast levels, respectively [29,30,31]. Scores were translated to logCS units for analysis, according to the VectorVision protocol [29,30,31].

Best-corrected contrast sensitivity was tested at a distance of 2.5m (8 feet) from the device. The participants were presented an illuminated suprathreshold example of the test pattern, selected by the examiner. The subjects were instructed to identify the test pattern between two targets presented on different rows [29]. The next level of spatial frequency was tested using the same protocol described above. Contrast threshold was scored according to the last column sine-wave target, correctly identified for each spatial frequency. This procedure was performed under three different lighting conditions: (1) photopic conditions, (2) mesopic conditions, and (3) mesopic conditions with induced glare.

The photopic conditions had the room lights turned off, and the luminance from the chart was 85 cd/m^2^. The mesopic conditions were simulated by subjects wearing a 1.5 neutral density filter whilst performing measurements. The glare was simulated using the machine inbuilt glare system, which is set at 75% of the maximum setting (52 lux). The measurements were performed in a randomized sequence.

#### 2.4.4. Blood Draw and Serum Analysis of Carotenoids 

Blood draws were performed by a licensed phlebotomist under sterile conditions with no infections or adverse reactions reported. The procedure was performed at baseline and at week 12 of the supplementation period. Separated serum samples were coded for anonymity and shipped to Eurofins Craft Technologies (Wilson, NC, USA) for analysis of the levels of lutein, zeaxanthin and *meso*-zeaxanthin. The carotenoids were analyzed by high-performance liquid chromatography. Lutein was separated from the combined zeaxanthin stereoisomers using a normal phase Chromegabond Diol 150 mm × 4 mm column with a 3 µm particle size. The mobile phase was 96% hexane/4% isopropyl alcohol (*v*/*v*) and detection was at 450 nm. The zeaxanthin fraction was collected and reinjected onto a Chiralpak AD, 4.6 × 250 mm with amylose tris (3,5-dimethylphenylcarbamate)-coated 5-µm silica particles. The mobile phase was 94% hexane/6% isopropyl alcohol (*v*/*v*) and detection was again at 450 nm. This technique separates the zeaxanthin stereoisomers from each other.

### 2.5. Statistical Analysis and Outcome Measures

Results are expressed for baseline and change after intake of supplementation as means and standard deviation for the serum levels of carotenoids, MPOD measures and CSF under various conditions. Two-tailed paired samples t-tests were utilized when evaluating changes from baseline within-group, and p-values of <0.05 were significant. A two-tailed independent samples t-test with unequal variances was utilized when evaluating between-group effects and p-values of <0.05 were significant. One-way repeated measures analysis of variance with Bonferroni correction was utilized to evaluate the changes in CSF between groups. Adjustments for potentially confounding factors such as body mass index were not included in the analyses. Change in CSF from baseline under various conditions was correlated with the change in measured MPOD using a Pearson correlation coefficient. 

## 3. Results

### 3.1. Carotenoid Uptake Analysis

Table 1 provides the mean and SD of all variables evaluated. The number of subjects that had completed this part of the study was 27, with 14 in the PV group and 13 in the LM group. The mean age of the participants of the LM group (24.57 SD 8.0 yrs) was not significantly different when compared to the PV group (30.6 SD 20.0 yrs) (independent samples t-test *p* = 0.30).

### 3.2. Lutein and Zeaxanthin Serum Analysis 

Table 1 provides the mean serum concentration of various carotenoids at baseline and at week 12 of the supplementation period in the LM group and the PV group. The baseline serum levels of lutein and zeaxanthin were 0.300 SD 0.174 and 0.112 SD 0.041 µmol/L, and 0.273 SD 0.097 and 0.123 SD 0.044 µmol/L, respectively, for the PV and LM group. The difference in the serum levels of carotenoids for both lutein and zeaxanthin at baseline were not significantly different between the groups (independent samples t-test *p* = 0.63 and 0.49).

There was an increase in the serum levels of lutein and zeaxanthin in both the PV group and the LM group. The serum levels of lutein and zeaxanthin at week 12 in the PV group were 0.400 SD 0.175 and 0.155 SD 0.039 µmol/L, which were 33% and 38% greater than the baseline values, respectively. The uptake of zeaxanthin was statistically significant (paired samples t-test *p* = 0.01). The increases in serum levels of lutein and zeaxanthin in the LM group were larger, especially for lutein. Compared to the baseline, the serum levels of lutein and zeaxanthin at week 12 were 0.705 SD 0.239 and 0.194 SD 0.045 µmol/L respectively, i.e., 158% and 58% greater than baseline (paired samples t-test *p* < 0.0001 and 0.0009). The difference in change of serum levels of carotenoids between the PV group and LM group was also different, with the LM group showing significantly greater uptake of lutein at week 12 (independent samples t-test *p* < 0.0001).

### 3.3. Meso-Zeaxanthin Serum Analysis

Table 1 provides the serum concentration of *meso*-zeaxanthin at baseline and at week 12 for the PV group and the LM group. The mean baseline serum concentration of mesozeaxanthin in the LM and the PV group was 0.0005 SD 0.0006 and 0.0002 SD 0.0004 µmol/L respectively. More than 64% of the participants had no detectable *meso*-zeaxanthin levels at baseline. Baseline analysis revealed that barely detectable levels of serum *meso*-zeaxanthin were seen in three subjects in the PV group and six subjects in the LM group. There was a large increase in the serum levels of *meso*-zeaxanthin at week 12 in the LM group and a very small increase in the PV group. The mean 12-week serum concentration of mesozeaxanthin in the LM and the PV group was 0.086 SD 0.048 and 0.001 SD 0.0006 µmol/L respectively (paired samples t-test *p* < 0.0001 and 0.0001 respectively). The difference in change of serum levels of *meso*-zeaxanthin between the PV group and LM group was also significantly different, with the LM group, not surprisingly, showing greater uptake of *meso*-zeaxanthin at week 12 (independent samples t-test *p* = 0.0005).

### 3.4. MPOD Change with Supplement Intake

Twenty-four individuals completed this part of the study, 12 in the LM group and 12 in the PV group respectively. Table 2 provides the mean and SD for the parameters evaluated in the study. The mean age of participants was not significantly different in the LM and the PV groups: 23.0 and 32.5 years respectively (independent samples t-test p-value 0.16). The mean MPOD and standard deviation (SD) for the PV and the LM groups at baseline were 0.46 (SD 0.27) and 0.42 (SD = 0.14), respectively, which were not significantly different (independent samples t-test *p* = 0.63). After a 24-week supplement intake period, the mean MPOD was 0.46 (SD 0.25) and 0.48 (SD 0.19) in the PV and the LM group respectively. There was a 15.2% change in mean MPOD at the end of the trial in the LM group, whereas the mean MPOD of the PV group was almost unchanged (−0.2%). While the LM group received a larger daily dose of total carotenoid than the PV group (28 vs. 12 mg), the increase in MPOD per milligram of total carotenoid was still significantly higher in the LM group compared to the PV group (independent samples t-test *p* = 0.024). It should also be noted that the MPOD increased for seven of the PV subjects, but decreased for the other five subjects. For those showing an increase, the average was 0.026. For the LM group, only one subject had a very small decrease in MPOD, and the average for the other 11 was 0.070. 

### 3.5. Change in Visual Acuity and CSF with Changes in MPOD

Twenty-four individuals completed this part of the study, 12 in the LM group and 12 in the PV group. Table 3 provides the mean and SD of the baseline logCS values and final logCS values at week 24 under all conditions. The mean age of participants was not significantly different in the LM and the PV group: 23.0 and 32.5 years respectively (independent samples t-test *p*-value 0.16). The baseline visual acuity and logCS were not significantly different between the LM group and the PV group (independent samples t-test *p* > 0.05). As seen from the table, there was an improvement in logCS at all spatial frequencies for both the LM group and the PV group. However, the change in logCS compared to baseline was not significantly different in both the PV group and the LM group in all measured spatial frequencies (paired samples t-test *p* > 0.05). The change in logCS was not significantly different when comparing between groups (one-way ANOVA with repeated measures F = 0.11, *p* = 0.74). 

Eleven out of twelve individuals in the LM group and 6 out of 12 in the PV group showed an increase in MPOD at the same time period. The changes in CSF under various conditions—photopic, mesopic and induced glare—were not significantly correlated with the observed changes in measured MPOD (Pearson correlation coefficients *p* > 0.05 for all correlations).

## 4. Discussion

The present study was designed to conduct a comparison of Lumega-Z (LM) and PreserVision (PV) AREDS-2, two commercially available nutritional supplements, albeit with different amounts of the retinal carotenoids. The goals of this a study were to fulfil the lack of clinically available comparisons of serum uptake, change in MPOD, and visual performance over time with these supplementations under ideal conditions. There are numerous differences in the constituents, contents and the type of delivery system between the supplements. The LM is a micronized, lipid-based, liquid carotenoid formula, whereas the PV is a softgel supplement. The levels of carotenoids, 12 vs. 28 mg for PV and LM respectively, and other constituents in the two supplements are also different. As the study aimed to be a “real-life” comparison of the products that are currently being recommended by doctors and taken by patients, attempts to equalize the levels of carotenoids or the delivery system were not made; however, to address this issue, we discuss and describe in the next paragraph the results after normalization based on the different milligram levels of ingredient concentrations in the two supplements. The constituents of LM and PV are provided in Appendix A.

As previously shown [9], this study proves that the serum levels of carotenoids show a rapid change with intake of the nutritional supplements, with a significant increase seen at week 12. It was indeed expected that the LM group would show a greater serum carotenoid uptake compared to the PV group, given that the level of both lutein and zeaxanthin, is 1.5 times greater in the LM supplement. The present study confirms that LM provides a higher uptake level than PV. While the changes in serum zeaxanthin for the two groups were consistent with the 1.5-fold difference in zeaxanthin content in the two supplements, the same was not true for serum lutein, where the LM group showed a greater than fourfold increase compared with the PV group, Overall, the increase in combined serum lutein and zeaxanthin was over three-fold higher for the LM group compared with the PV group. These results indicate overall better bioavailability of the LM supplement compared with the PV supplement. The difference in serum uptake in these groups may be in part due to the difference in the delivery systems of the two carotenoids. However, cumulative synergistic effects due to the other additional constituents in LM supplement cannot be discounted, and this requires further investigation.

*Meso*-zeaxanthin is an isomeric conversion of lutein in the retina through RPE65 isomerase [32] although there appear to be food products, including chicken eggs from California and Mexico, that contain small amounts of *meso*-zeaxanthin. A review is provided by Nolan et al. [33]. This could, in principle, account for the presence of correspondingly very small amounts of *meso*-zeaxanthin in the baseline serum samples of certain subjects. The LM supplement has *meso*-zeaxanthin (10 mg) whereas the PV supplement does not report having any *meso*-zeaxanthin. It is interesting to note that, despite the absence of reported *meso*-zeaxanthin in the PV supplement, a very small increase in serum levels was noted at week 12. In a study undertaken by Prado-Cabrero et al. [34], it was shown that nutritional supplements often did not report having *meso*-zeaxanthin but contained detectable amounts when examined using high-performance liquid chromatography. Although it should be noted that PV showed undetectable levels of *meso*-zeaxanthin in their study [34], five other supplements using lutein from the same source, Floraglo^®^ Lutein, showed amounts ranging from 0.04 to 0.94 mg. Further testing is warranted in this area, as the data obtained in the present study would indicate that the PV supplement may have *meso*-zeaxanthin, albeit in a very small amount.

The PV supplement, which mirrors the formulation of the treatment ingredients used in the AREDS-2 trial, has been widely investigated for its use in individuals with AMD [22]. The trial showed that the PV ingredients helped in risk-reduction and prevented progression from moderate to advanced AMD and found that the benefits of lutein and zeaxanthin were most visible in individuals that had lower baseline levels of the serum carotenoids. Furthermore, it showed that lutein and zeaxanthin may provide benefits beyond other ingredients, as these could be a safer alternative to beta-carotene—which was found to increase the risk of development of lung cancer in smokers [22]. Like our study, the AREDS-2 trial evaluated the serum levels of lutein and zeaxanthin. That trial found an increase in serum levels of lutein and zeaxanthin at years 1, 3 and 5 [22]. The current study provides evidence that the PV supplement does indeed increase serum carotenoid levels significantly after only 12 weeks. For MPOD, although approximately 60% of subjects showed a trend toward an increase, there was no significant increase on average in MPOD level in the PV group, even at week 24. Unfortunately, the AREDS-2 study did not test MPOD, suggesting further study is needed to establish that a longer treatment duration with PV may eventually lead to a statistically significant elevation of MPOD levels.

In comparison to the PV group, the LM group showed significantly greater serum concentrations at week 12 and increased MPOD at week 24. Given that we know that the dose–time relation of carotenoid is an important predictor of visual function increase, it could be hypothesized that an increased level of carotenoid intake may prevent progression of mild-stage AMD to moderate-stage AMD. Future randomized control trials could evaluate the benefits, investigating whether higher intake and bioavailability of carotenoids leads to risk reduction for AMD progression.

MPOD, as measured by hetereochromatic flicker photometry, showed a significant increase at week 24 for the LM group, whereas the PV group did not show any change on average. This could be in part due to the greater amount of carotenoid and possibly in part due to a greater bioavailability in the LM supplement. In a meta-analysis performed by Liu et al. [35], it was shown that the MPOD is indeed elevated with intake of carotenoids, and that it is both a function of dose and time response in a healthy population and in individuals with AMD. The present study adds to the large body of evidence that MPOD can indeed by augmented using nutritional supplements.

This study also evaluated changes in visual function as measured using CSF under various viewing conditions: photopic, mesopic and mesopic with induced glare. It has been shown that MPOD can be augmented with dietary increase, and this results in improved visual function [1,16,17,18,19,20,21,35,36,37]. This result could be because, along with its protective function, the carotenoid concentration in the macula serves to filter out the effects of chromatic aberration in the eye [1,16,17,18,19,20,21,35,36,37]. Our study did not show an improvement in CSF with increase in MPOD at week 24. This could be in part because of the relatively short duration of the study and in part because our study group consisted of individuals in good ocular health with normal contrast sensitivity and not at risk of AMD. These findings are in agreement with those of a previous randomized placebo-controlled trial [38]. After 24 weeks of carotenoid supplementation, Ma et al. [38] found measurable MPOD changes; however, the CSF did not change significantly during the same time period. When carotenoid supplementation was continued to week 48, visual performance as measured by CSF was improved overall.

This study is the first to provide a head-to-head comparison of two commercially available supplements, PV with 12 mg of L and Z and LM with 28 mg of L, Z and MZ, with regard to the changes in serum levels of carotenoids, visual function and MPOD. The study results show that higher concentration of carotenoids indeed translates to a greater elevation of carotenoids in the target tissues in a healthy population. At this stage, we are unable to attribute the larger increase in MPOD seen in the LM group to any one carotenoid, such as MZ, since individual carotenoids cannot be measured in the living eye. Subsequent studies will need to evaluate the changes in individuals at risk of AMD. AMD remains the leading cause of blindness, and progress in treatment modalities of neovascular AMD has been by leaps and bounds, where the goal is not just maintenance of vision but rather improvement of vision. However, progress in treatment modalities of non-exudate AMD, which is the more common variety of AMD, seems to elude us, and at best aims towards maintaining vision rather than improving it. With an increase in our understanding of carotenoids, optimization of their speed of uptake, and development of technology that measures MPOD quickly and accurately, we can hope that improvement of visual function will be the new endpoint or goal of all AMD therapies.

## Figures and Tables

**Table 1:** Serum carotenoid levels during the study period.

	LumegaZ™ Group N = 14		Change within Group Comparison to Baseline	PreserVision™ Group N = 14		Change within Group Comparison to Baseline	Between Groups Comparison of Baseline	Between Groups Comparison of Change Over 12-Week Period
Mean (SD)	Baseline	12-Week	*p*-Value	Baseline	12-Week	*p*-Value	*p*-Value	*p*-Value
Lutein	0.273	0.705	<0.0001	0.300	0.400	0.157	0.63	<0.0001
	(0.097)	(0.239)		(0.174)	(0.175)			
Zeaxanthin	0.123	0.194	0.0009	0.122	0.155	0.01	0.49	0.29
	(0.044)	(0.045)		(0.041)	(0.039)			
*Meso*-zeaxanthin	0.0005	0.086	<0.0001	0.0002	0.001	0.0001	0.11	0.0005
	(0.0006)	(0.048)		(0.0004)	(0.0006)			

Lutein, zeaxanthin and meso-zeaxanthin measured in µmol/L. Within-group comparison: two-tailed, paired samples t-test. Between-group comparison: two-tailed, independent samples t-test with unequal variance.

**Table 2:** Macular pigment optical density measurements during the study period.

Mean (SD)	LumegaZ™ Group N = 12		Change within Group Comparison to Baseline	PreserVision™ Group N = 12		Within Group Comparison to Baseline	Between Groups Comparison of Baseline	Between Groups Comparison of Change Over 24-Week Period
	Baseline	24-Week	*p*-Value	Baseline	24-Week	*p*-Value	*p*-Value	*p*-Value
MPOD	0.418	0.482	0.019	0.460	0.459	0.926	0.63	0.024
	(0.15)	(0.19)		(0.26)	(0.25)			

MPOD is macular pigment optical density as measured using the Mapcat SF™ heterochromatic flicker photometer. Within-group comparison: two-tailed, paired samples t-test. Between-group comparison: two-tailed, independent samples t-test with unequal variance.

**Table 3:** Contrast sensitivity function as measured under various conditions.

Mean (SD)		LumegaZ™ Group N = 12								PreserVision™ Group N = 12							
		Baseline				24-Week				Baseline				24-Week			
LogMAR Visual acuity		−0.040 (0.09)				−0.028 (0.07)				+0.035 (+0.18)				+0.020 (0.17)			
Contrast sensitivity in cycles per degree (CPD)		3	6	12	18	3	6	12	18	3	6	12	18	3	6	12	18
	Photopic	1.64	1.8	1.57	1.14	1.74	2.03	1.62	1.24	1.59	1.75	1.40	0.92	1.71	1.86	1.56	1.10
		(0.11)	(0.14)	(0.22)	(0.28)	(0.14)	(0.23)	(0.21)	(0.18)	(0.20)	(0.31)	(0.22)	(0.33)	(0.15)	(0.30)	(0.30)	(0.27)
	Mesopic	1.41	1.49	1.01	0.56	1.61	1.64	1.23	0.87	1.43	1.38	0.82	0.45	1.58	1.59	1.14	0.77
		(0.26)	(0.25)	(0.31)	(0.26)	(0.22)	(0.29)	(0.27)	(0.38)	(0.23)	(0.25)	(0.30)	(0.30)	(0.27)	(0.26)	(0.30)	(0.33)
	Glare	1.50	1.52	1.03	1.66	1.57	1.68	1.09	0.77	1.46	1.28	0.94	0.56	1.60	1.57	1.08	0.78
		(0.13)	(0.37)	(0.38)	(0.43)	(0.20)	(0.34)	(0.26)	(0.27)	(0.21)	(0.31)	(0.33)	(0.29)	(0.26)	(0.31)	(0.30)	(0.32)

Age, LogMar visual acuity at baseline was not significantly different (*p* = 0.16 and 0.22 respectively). Change in visual acuity after a 24-week period was not significantly different compared to baseline either within the group (*p* = 0.64 and 0.44 respectively) or between groups (*p* = 0.40). Changes in log contrast sensitivity to the baseline were not significantly different at all spatial frequencies when comparing within groups (all paired samples tests *p*-values > 0.05). Changes in log contrast sensitivity from baseline were not significantly different at all spatial frequencies, when comparing between groups (one-way ANOVA with repeated measures F = 0.11, *p* = 0.74).

## Appendix A

**Table A1:** Constituents of Lumega-Z and PreserVision AREDS2 supplement.

Formula Products	Guardion Health Sciences Lumega-Z	Bausch + Lomb PreserVision AREDS 2 Formula
**Vitamin C**	500 mg	500 mg
**Thiamin**	1.5 mg	
**Riboflavin**	1.7 mg	
**Niacin**	20 mg	
**Vitamin B6**	10 mg	
**Folate**	800 mcg	
**Vitamin B12**	1000 mcg	
**Vitamin D3**	2000 IU	
**Vitamin E**	200 IU	400 IU
**Vitamin K**		
**Biotin**	100 mcg	
**Pantothenic Acid**	10 mg	
**Calcium**	250 mg	
**Iodine**		
**Magnesium**	100 mg	
**Zinc**	25 mg	80 mg
**Selenium**	70 mg	
**Copper**	3 mg	2 mg
**Manganese**	2 mg	
**Chromium**	120 mcg	
**Molybdenum**	75 mcg	
**NAC**	500 mg	
**POA Blend**	200 mg	
**Acetyl-L-Carnitine**	500 mg	
**Taurine**	500 mg	
**Quercetin**	100 mg	
**CoQ10**	50 mg	
**Lycopene**	500 mcg	
**Lutein**	15 mg	10 mg
**Zeaxanthin**	3 mg	2 mg
**Meso-Zeaxanthin**	10 mg	
**Astaxanthin**	4000 mcg	
